# Intra-group decision-making in agent-based models

**DOI:** 10.1038/s41598-021-96661-5

**Published:** 2021-09-06

**Authors:** Allegra A. Beal Cohen, Rachata Muneepeerakul, Gregory Kiker

**Affiliations:** Department of Agricultural and Biological Engineering, University of Florida, 1741 Museum Road, PO Box 110570, Gainesville, FL 32611-0570 USA

**Keywords:** Mathematics and computing, Computational science

## Abstract

Many agent-based models (ABMs) try to explain large-scale phenomena by reducing them to behaviors at lower scales. At these scales in social systems are *functional groups* such as households, religious congregations, coops and local governments. The *intra-group dynamics* of functional groups often generate inefficient or unexpected behavior that cannot be predicted by modeling groups as basic units. We introduce a framework for modeling intra-group decision-making and its interaction with social norms, using the household as our focus. We select phenomena related to women’s empowerment in agriculture as examples influenced by both intra-household dynamics and gender norms. Our framework proves more capable of replicating these phenomena than two common types of ABMs. We conclude that it is not enough to build multi-scale models; explaining social behaviors entails modeling *intra*-scale dynamics.

## Introduction

Between individuals and societies are *functional groups* of vastly different scales, such as governments, workplaces, churches and households. Members of groups interact with each other differently than with strangers. First, members of groups depend on each other through shared resources and responsibilities. For example, members of a community center keep the center in good repair and do each other small favors. Second, group members have histories with each other that they do not with random individuals, such as long-standing deals between community center members about volunteer scheduling. Third, members of groups must often make *joint decisions* about how to spend resources, assign responsibilities, and respond to social norms and disturbances. For instance, community center members might decide whether to donate parking lot space to a mobile COVID-19 testing center.

When making joint decisions, group members bring heterogeneous preferences and beliefs that can create conflict. These preferences and beliefs are often influenced by social norms; for example, some community center members might think COVID-19 is a hoax. The conflict and negotiation inherent in intra-group decision-making can generate unexpected or inefficient outcomes at the group scale. Moreover, these outcomes can spread to other social structures and influence social norms at larger scales; for example, if one community center minimizes the importance of COVID-19 testing, so might another, which might spread regional beliefs that COVID-19 is a hoax.

If one wants to predict and explain how large-scale social dynamics emerge from behaviors at multiple scales, one needs to model behavior between scales (e.g. how social norms interact with community decisions) *and* within scales (e.g. how community members decide whether to host a testing center). This entails modeling not only functional groups, but intra-group dynamics. However, even multi-scale agent-based models (ABMs) tend to overlook dynamics that happen within groups^1–4^. This paper presents a framework for modeling intra-group decision-making as well as the interactions between individuals, groups and social norms.

**Figure 1:**
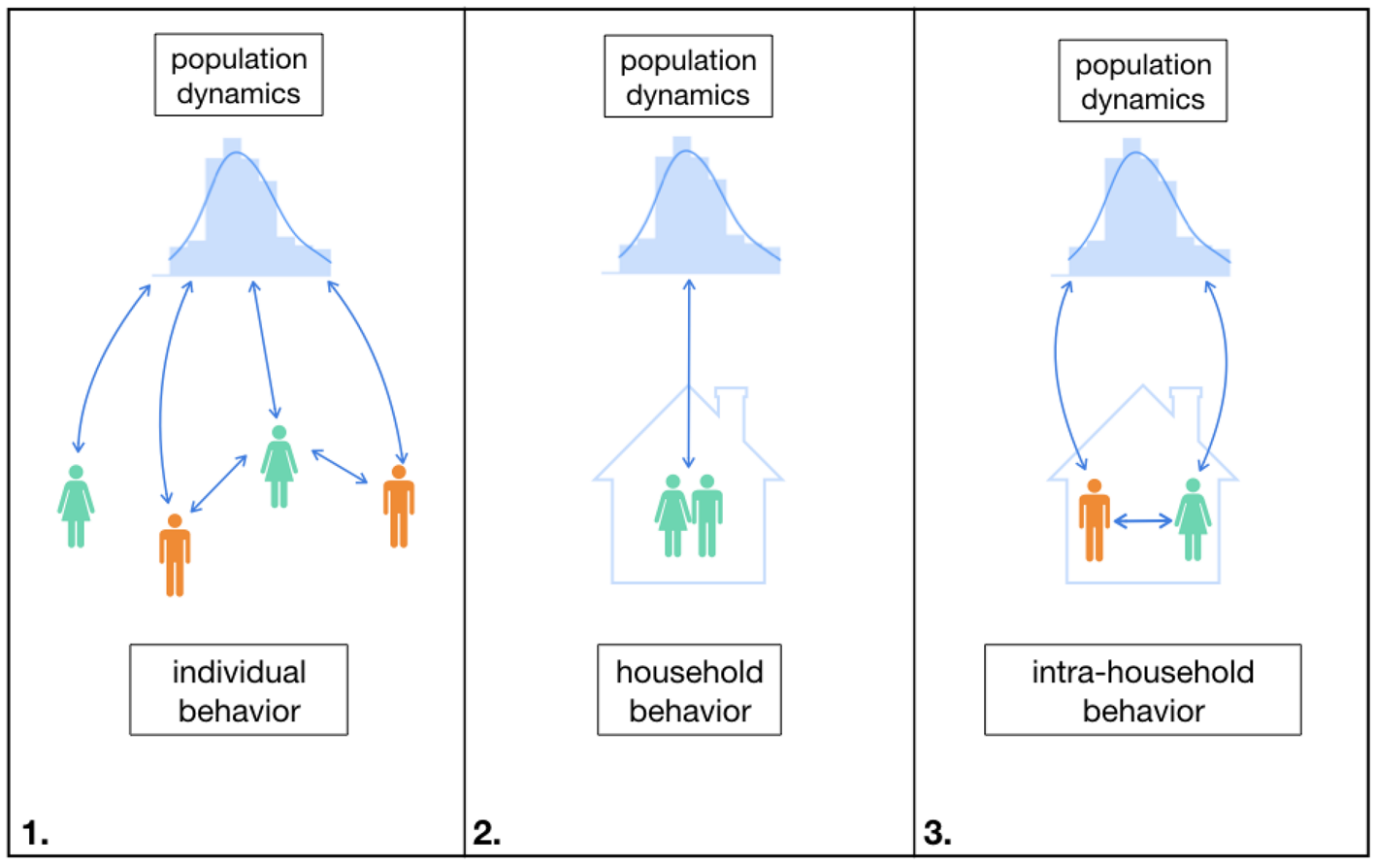
Differences between the individual, unitary and intra-household frameworks. Arrows indicate interaction, the colors of the agents represent some set of preferences (i.e. “green” is different than “orange”.) Panel 1 shows an individual-based ABM of social norms in which agents have different preferences, interact with one another and with social norms, but do not interact as household members. Panel 2 shows a unitary household-based ABM with a single set of preferences and no intra-household interaction. Panel 3 shows our ABM with intra-household decision-making between heterogeneous agents.

Our focus will be intra-household decision-making. The household is the site of decision-making about a long list of phenomena—elder care, child nutrition, the number of children a woman has, vaccination, the acceptance of queer children, the passing of land between generations, intimate partner violence, and the spread of COVID-19 among roommates, to name a few. The household is also a particularly compelling functional group because decision-making is influenced both by within-group factors, such as the heterogeneous preferences of household members, and by dynamics at higher scales, such as social norms. Though individual-individual interaction occurs in many individual-based ABMs (Panel 1 of Fig. 1), joint decision-making—such as that within households—does not. Here let us clarify that there are household-based ABMs^2,3,5–7^, (Panel 2 of Fig. 1) but they overlook the interactions between household members. These models treat the household as *unitary*, assuming that decisions are made by a single household head using one set of preferences^5^. In addition to treating household members as homogeneous, unitary household-based ABMs overlook the different effects of social norms on household members; for example, gender norms in agricultural households contribute to production inefficiency that is not predicted by the unitary view of the household^8^.

To introduce our framework, consider individual versus joint decision-making about how much time to spend working and cleaning one’s house. An individual-based ABM might define a person’s utility for Activity 1 (working) and Activity 2 (cleaning) as $p_1, p_2$, and split the person’s time between each activity, represented by functions $f_1(), f_2()$ that take time and return goods:

1 $$\begin{aligned} p_{1} \cdot f_1(t_{1}) + p_{2} \cdot f_2(t_{2})\text { s.t. } t_1 + t_2 = 1 \end{aligned}$$

Different people may value the payoffs of each activity differently (Person A prefers a clean house, Person B prefers money in the bank) and receive different payoffs (A is paid less than B). At no point, however, do Person A and Person B make a joint decision about how to spend their collective time. This approach, which we shall call the *individual framework*, is common in agent-based modeling and works well when the primary goal is to investigate the relationship between individuals and entities larger than individuals (e.g. individuals and social norms)^9–13^.

Without joint decision-making between agents, concepts like specialization (“We’ll split these tasks based on our skills”) and coercion (“You’ll do that task because I make you”) become cumbersome to model. A second approach in agent-based modeling is to combine the resources of multiple group members that share the same set of preferences, such as the time of Person A and B:

2 $$\begin{aligned} p_{1} \cdot f_1(t^A_{1} + t^B_{1}) + p_{2} \cdot f_2(t^A_{2} + t^B_{2})  \text {s.t. } t^A_{1} + t^A_{2} = 1, t^B_{1} + t^B_{2} = 1 \end{aligned}$$

We shall call this the *unitary framework*, after Becker’s 1965 theory of the unitary household^5^. The defining aspect of this framework is that members are homogeneous and cooperate perfectly, without any opportunity for negotiation^2,3,5–7^. This makes modeling phenomena like inequity (members must have their own personal resources) and trust (members must be able to disagree and act uncooperatively) challenging if not impossible.

Suppose instead that A and B were part of the same group, but had different preferences. How would they resolve disagreements about how to spend their time? One mechanism might be *trading* and *sharing* the goods produced by private ($f_1()$) and public ($f_2()$) activities. A divides her time between two activities with different payoffs and B does the same. But now they *share* one type of payoff ($f_{2}(t^A_{2} + t^B_{2})$) and *trade* the other ($\theta \cdot f^A_1(t^A_{1})$):

3 $$\begin{aligned} p^A_{1} \cdot [f^A_1(t^A_{1}) \cdot (1 - \theta )] + p^A_{2} \cdot f_{2}(t^A_{2} + t^B_{2}) \end{aligned}$$

4 $$\begin{aligned} p^B_{1} \cdot [f^B_1(t^B_{1}) + f^A_1(t^A_{1}) \cdot \theta ] + p^B_{2} \cdot f_{2}(t^A_{2} + t^B_{2}) \end{aligned}$$

For example, when A cleans the house, B can enjoy it (sharing a *public good*); and when B goes to work, she can give some $\theta$ of her wages to A (trading a *private good*). Moreover, we can model the *bargain* that emerges, e.g. A cleans the house more often *because* B gives her more money, and even the potential power imbalances that tip the bargain one way or the other. We shall call this the *intra-household framework*, in which A and B make joint decisions within the household as well as interact with the broader community (Panel 3 in Fig. 1). As optimization problems, the individual and unitary frameworks are straightforward, but the intra-household framework is more complicated. Not only does the intra-household framework have two heterogeneous and dependent equations, it requires a *decision-making protocol* for reconciling them (or a *bargain*). However, the intra-household framework looks a lot more like the reality of interaction and decision-making within households. In fact, models of intra-household decision-making are well established in economics, but they are largely theoretical and have neither the expressivity nor the dynamic social mechanisms of ABMs^14–18^.

In this paper, we introduce an agent-based model with an intra-household bargain and a mechanism for social norms, abbreviated as “IHM” (intra-household model). Table 1 shows the abbreviations used throughout the paper. We also build comparison models from the individual and unitary frameworks, abbreviated as “IM” and “UM” respectively (individual-based and unitary household-based models). We ask whether IHM produces qualitatively different behaviors than IM and UM. While some differences are already formally evident at the household scale from Eqs. (1), (2), (3), (4), the effects of social norms in the IHM, IM and UM models cannot be fully anticipated. Thus, it is necessary to use simulation to explore and compare emergent behavior between the three models.

We select gender inequity in agriculture as our example domain for two reasons. First, it is an important problem: Women are crucial to ensuring the food security of a growing global population. The FAO estimates that providing female farmers with access to resources could reduce the number of undernourished people in developing countries by 100 to 150 million^19^. Additionally, when women control resources, they tend to allocate more towards their children, which can lead to more productive households in the future^20–26^. Second, gender and development literature makes it clear that cultural norms and intra-household dynamics cannot be treated separately when evaluating strategies for empowering women^27–29^. For example, one strategy for empowering women in agriculture is to increase their autonomy in income. This can theoretically be achieved by making private activities more lucrative for women, but in practice women are subject to the approval of their husbands and communities^30^. Therefore, a pertinent question is, “How high do women’s wages have to be for women to enter the workforce, given the influence of their households and gender norms?”.

To compare how IHM, IM and UM address this question, we create a simple example of two activities (private and public). IM agents allocate time to each activity individually; UM agents allocate time according to a shared set of preferences as a household; and IHM agents allocate their time according to a household bargain where private good is traded and public good is shared. The private activity returns separate wages $w_f, w_m$ for female agents and male agents respectively; the public activity returns one unit of public good per unit time. Agents are split into two norm populations based on gender, and norm populations are initialized according to gender roles: Female agents begin the simulations doing mostly public activity (“housewives”) and male agents begin the simulations doing mostly private activity (“breadwinners”). Agents of both genders are parameterized with heterogeneous goods preferences (e.g. Agent A prefers private good slightly more than public good, Agent B prefers public good much more than private good) as well as levels of conformity (e.g. Agent A prefers to conform to her social norm much more than she likes to receive private good), although we note again that UM agents act under a single set of preferences according to the unitary framework. The mechanism behind social norms is described in detail in “Norms” section; for now it is enough to know that agents strive to reduce the distance between themselves and the average behavior of their norm population according to the strength of their conformity parameters.

After the initialization of simulations, we increase the private activity wage of female agents ($w_f$) while holding male wage constant and examine the change in time spent on private activity for both male and female agents. We simulate increases in $w_f$ under two parameterization conditions. “CM” refers to the condition in which male agents are highly conformist, i.e. they subscribe heavily to social norms and may even prefer to conform at the expense of public or private good. “MP” refers to the condition in which all agents have moderate preferences and conformity, i.e. their preferences for public and private good are not much larger or smaller than their preference for conforming to gender norms.

The details of our intra-household implementation can be found in “Overview of the intra-household model” section, while the details of IM and UM can be found in “Comparison models” section. Further description of the simulations and parameterization conditions can be found in “Simulation experiments” section.

**Table 1:** Glossary of terms.

Abbreviation	Term
IHM	Intra-household model (Panel 3 in Figs. 1; 2)
IM	Individual-based model (Panel 1 in Fig. 1)
UM	Unitary household-based model (Panel 2 in Fig. 1)
CM	Conformist males with moderate preferences
MP	Moderate preferences and conformity
$w_f$	Wage paid to female agents doing private activity (varied from 0.1 before $t = 25$ to multiple values)
$w_m$	Wage paid to male agents doing private activity (fixed at 0.6)

## Results

The y-axis of Fig. 3 shows the total change in time spent by female and male agents on the private activity (wage work) from the time step after the wage increase to the end of the simulation. The x-axis shows post-change $w_f$, and the plots are organized by agent gender and experimental condition.

**Figure 2:**
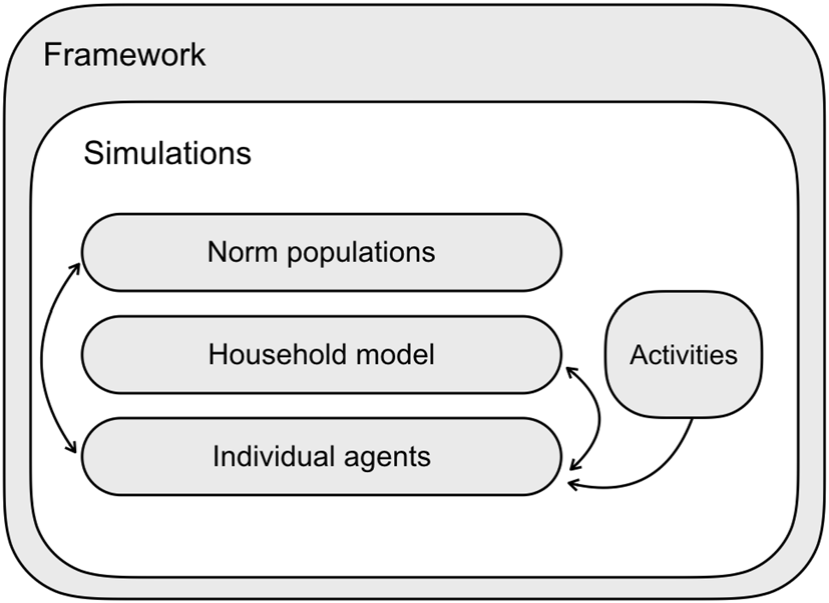
A diagram of the structure of the framework. Arrows indicate interaction.

**Figure 3:**
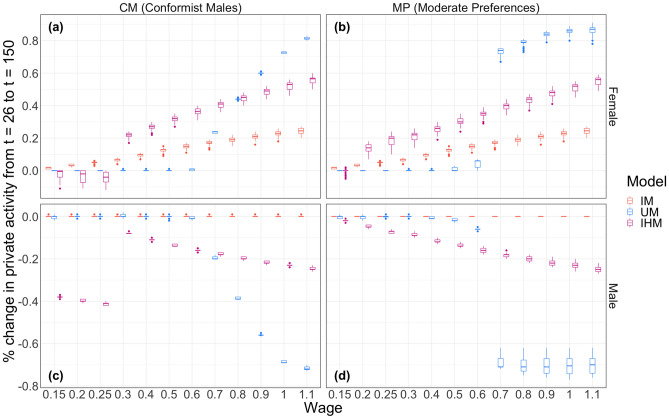
Change in time spent on private activity by agents from $t = 26$ (after wage change) to $t = 150$ (simulation end) under different wage change conditions. Colors indicate the model used in the simulation, i.e. IHM (intra-household model), IM (individual-based model) or UM (unitary household-based model). Panels refer to the agents and conformity condition: (**a**) Female agents’ behavior under the CM condition; (**b**) female agents’ behavior under the MP condition; (**c**) male agents’ behavior under the CM condition; and (**d**) male agents’ behavior under the MP condition.

First, we note the expected qualitative behavior of the models as confirmed by the simulations. Generally, the IM results are less sensitive to wage increases than UM and IHM. In Fig. 3a,b, female IM agents adjust their behavior almost linearly in response to increasing $w_f$, with a Pearson’s correlation of 0.98 (N = 1198, $p < 2.2e^{-16}$). This is because, with the exception of edge cases where agents have zero preference for one type of good, IM agents always lose some utility from one good by doing more of the other activity. The variance in female IM agents’ behavior change also increases roughly linearly with $w_f$. This is due to variation in the agents’ goods preferences. As $w_f$ increases, agents who have higher private good preference will adopt more private activity than agents who have higher public good preference (i.e. small increases in $w_f$ are worthwhile for everybody, but agents have different preference thresholds after which an increase in private activity is not beneficial).

Compared to the IHM results, IM shows more change in female agent behavior at low $w_f$ and less change at higher $w_f$, as seen in Fig. 3a,b. IM agents make small adjustments in response to wage changes because an increase in one type of good necessarily causes a decrease in the other type. UM and IHM agents, however, can pool their time with their spouse to preserve equal or greater levels of private and public good in response to external changes. This coordination allows for larger total behavior changes than are seen in the IM results. This is the case except for low $w_f$, where both UM and IHM agents are constrained by household decision-making while IM agents are not, leading to comparatively larger behavior changes by IM agents. In Fig. 3a,b, IM female agent variance is smaller than IHM for larger $w_f$ because IHM agents pool their resources and account for two sets of preferences, generating a wider set of behavior. Trivially, we note that male IM agents in Fig. 3c,d do not respond to changes in $w_f$ at all, because male and female agents are not linked by households. Likewise, female IM agent behavior does not vary between experimental conditions in Fig. 3a,b, because female agents are not affected by the level of male conformity.

Next, we note that UM systematically yields lower or higher levels of behavior change than IHM results, but the UM results are consistently centered around $w_f = w_m = 0.6$. The UM results are enormously sensitive to whether $w_f > w_m$; in Fig. 3a,b, female agents transition suddenly into the workplace once $w_f > 0.6$, while male agents in Fig. 3c,d take up housework. This is because, with two sets of resources to allocate and one utility function, the UM optimization problem sends agents to work in whichever manner maximizes payoff. In our simulations, this depends solely on which agent makes more money. While the threshold effect of the UM results is tempered by conformity (i.e. households with highly conformist males in Fig. 3a,c respond less to changes in $w_f$), the results still indicate that wage equality must be achieved before female agents can work outside the household in any meaningful capacity.

Though the qualitative behavior of the models is for the most part expected, the simulations also produced some unexpected emergent behavior, particularly in the IHM results. First, we note that the point at which female IHM agents change their behavior in response to $w_f$ varies between experimental conditions in Fig. 3a,b. That is, the increase in $w_f$ required to send female agents to work is greater in the CM condition than in the MP condition. This is not the case for either UM or IM. While the UM results do vary with experimental condition, the point at which female agents transition to the workforce is fixed at $w_f$; in the IHM results, that point is sensitive to the attributes of the male spouses.

Male IHM agents show some unexpected behavior at $w \le 0.25$ in Fig. 3c. While both IM and UM male agents do not change their behavior at all at when $w_f \le 0.25$, IHM male agents reduce the amount of time they spend working quite drastically. In fact, they change their behavior more at $w_f \le 0.25$ than at $w_f = 1.1$ ($p < 2.2e^{-16}$, Welch’s t-test). Likewise, in Fig. 3a the IHM female agents at $w_f \le 0.25$ experience a drop in time spent working outside the home.

**Figure 4:**
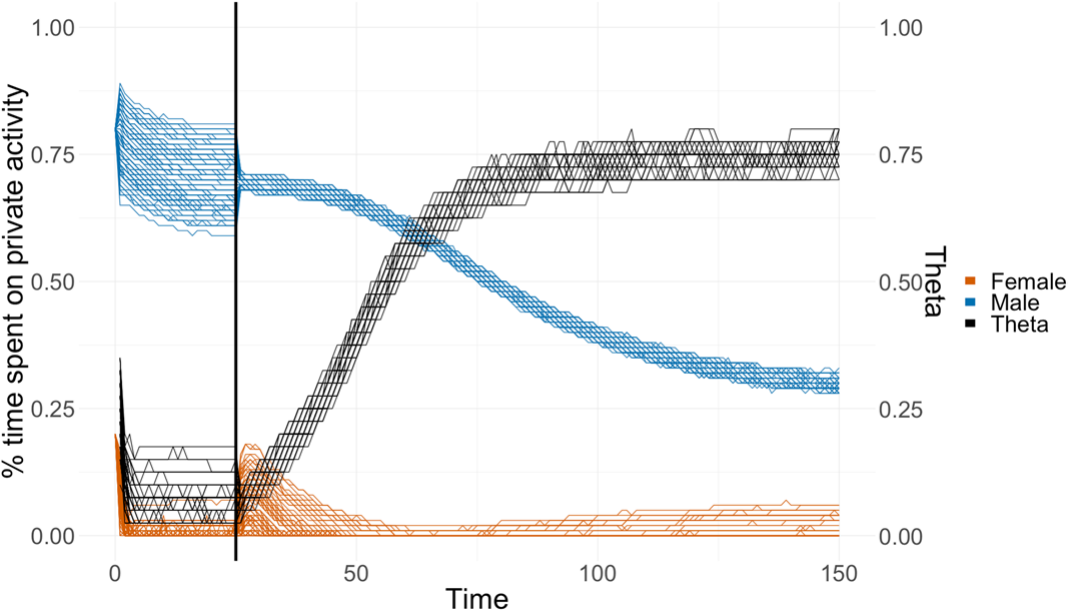
Time series of one simulation run with the IHM under the Conformist Males condition with a wage change of $w_f = 0.2$, marked with a vertical line at $t = 25$. The y-axis shows the percentage of private activity being done by female agents (red) and male agents (blue), in addition to $\theta$ (the percentage of private good being traded between agents within households, in black). Individual red and blue lines correspond with single agents, while each black line represents the $\theta$ of one household (i.e., agent pair).

This unexpected response at $w_f \le 0.25$ in Fig. 3c is a result of the male agents’ high conformity parameters. As described in “Overview of the intra-household model” section, agents conform more or less strongly to their population norm; in these simulations, the norm is simply an average of agent behavior updated at each time step. Male agents want to reduce the distance between their own behavior, e.g. time spent on private activity, and the moving average of all male agent behavior. When male agents’ conformity is higher than their interest in private and public goods, conditions can exist in which conforming to the average behavior gives male agents more utility than satisfying goods preference, as when $w_f \le 0.25$ in Fig. 3c. The result is a snowball effect leading to a total negative change in private activity done by male agents in simulations where $w_f \le 0.25$. This effect can be seen in more detail in the time series plot in Fig. 4, showing an IHM simulation where $w_f = 0.2$. At $t = 25$, $w_f$ is increased to 0.2 and male conformity is increased drastically (see “Simulation experiments” section for details). In the earliest time steps after the wage change, male agents make small initial adjustments to trading and sharing, namely increasing $\theta$ to keep female agents at home and working slightly less outside of the home to keep the level of public good desirable. Female agents begin to increase their private activity levels. However, male agents continue to spend less and less time working outside the home, and continue to increase the private good transferred to female agents, which causes the female agents to reduce their private activity time as well (Figs. 3a and  4). This pattern is not seen when $w_f > 0.25$ because the payoff of female agents’ private activity is larger than the potential payoff of reducing distance from the norm, even for conformist males. It is important to emphasize that the results at $w_f \le 0.25$ are not just due to the conformity of the male agents but to the small initial adjustments made to satisfy the intra-household bargain between male and female agents. No such bargain exists in the UM and IM frameworks.

## Discussion

The qualitative findings presented here show that IM and UM cannot replicate IHM results either on a basic level or through emergent behaviors. To understand why this matters, it helps to orient our results in our real-world context. Here we will discuss our findings in the context of several gender in agriculture case studies to show why modeling intra-group decision-making in ABMs is necessary for examining social problems.

The shortcomings of IM and UM can be described as a lack of cooperation and a lack of conflict, respectively. The gender and development literature makes it especially clear that it is useless to model one without the other. Even if one’s focus is on social norms, such as norms about which crops women can grow and who in the household is responsible for schooling costs, these tend to change as a result of adjustments made within the household. Consider Richard Schroeder’s 1999 account of the Gambian gardening boom, *Shady Practices*, which examined changing intra-household dynamics as women began to spend more time tending high-value gardens. As women spent less time on domestic duties and saw their incomes increase, men’s incomes decreased due to drought. The intra-household bargaining over time and money gave rise to a change in norms, in which men stopped undermining women’s efforts and began to help more around the house^31^. IM cannot replicate the cooperation that was eventually achieved, where women were able to specialize in gardening as men picked up the slack at home. When $w_f > w_m$, for instance, IM showed a smaller total change in female behavior than IHM, as well as a slightly more gradual relationship between wage increase and behavior change. Female IHM agents were able to make larger adjustments as a result of their ability to maximize two sets of resources.

On the other hand, the UM framework cannot replicate the conflict seen at the beginning of the Gambian gardening boom, where household welfare suffered as men avoided domestic work and women developed several different strategies to use their income to “buy goodwill” from their husbands (p. 49^31^). In fact, UM indicates that the transition of women from homemakers to wage earners will follow whatever path is most efficient, i.e. a threshold effect oriented around $w_f = w_m$. There is no evidence that wage equality is always the sole driver of household dynamics. For example, even when Gambian women were making vastly more money than men in the gardening boom, they found themselves undermined not only by social norms about land ownership and marketing but by their own husbands^31^. IHM results, by contrast, are sensitive to the conflict created by different spousal preferences, accounting through $\theta$ for the cost of disagreements, which results in female agents joining the workforce at different $w_f$ depending on the attributes of male agents.

Further examination of the gender and development literature reiterates the need for intra-group modeling. In the unitary framework, the household pools its resources and acts efficiently. However, empirical households do not always pool their resources and are not always efficient^32,33^. For example, Udry et al. found that in Burkina Faso households, the application of resources to agricultural plots was so unequal that a simple reallocation of fertilizer would improve women’s yields without any loss of production for men’s fields^8^. Many studies since have shown that resources do not always enter a common pool in the household to be used efficiently and that resource allocation depends at least in part on the identity of the allocator (usually men)^34^. UM cannot replicate this kind of inefficiency, which stems from a combination of patriarchal social norms and low bargaining power for women. However, we saw that IHM is capable of replicating inefficient states, such as when $w_f < 0.25$ in the CM condition. Male agents in pursuit of the norm transitioned their households into an inefficient mode of operation where more time was spent on the public activity than optimal and more private good was transferred from male to female agents than necessary.

Similarly, Jones’ study of the SEMRY rice irrigation project in 1983 details the ways in which households in Cameroon divide, share and trade inputs and outputs rather than pooling them. In particular, she noted that women often withheld labor inefficiently unless compensated through wages by their husbands, and that control over land and the product of labor was a point of contention in most households as was the spending of capital (i.e. not all household members shared the same preferences). While our experimental scenario was simple, the parallels are clear in the IHM results: Female agents stay home to work when male agents can afford their wages, but invest their time in private activities when the offered wages are too low. The UM framework cannot replicate these findings because agents are assigned whichever levels of activity make the household efficient according to one set of preferences.

Our implementation has its own shortcomings: It does not support functional groups larger than two-person households, such as farmer cooperatives and governments, and it overlooks common household arrangements such as polygamy. At the agent level, our model does not simulate the more nuanced aspects of decision-making such as satisficing or habitual behavior^35^. While utility functions can be designed to capture these patterns, the process may not be easy and the computational cost of more complex decision-making can be high. As we expand our implementation to N-person groups, we want to strike a balance between expressivity and parsimony, which may entail alternative modeling methodologies. We have identified several algorithmic approaches to decision-making to explore in our future work: Game theoretic methods like Minimax; weighted fair share algorithms such as those used for allocating computing resources; and probabilistic approaches to individual and group decision-making. Not all of these approaches are designed specifically for intra-group decision-making, but most have been used successfully for related problems^36–38^. Interested researchers may find the code for our current implementation here https://github.com/Allegra-Cohen/intrahouseholdmodel.

The goal of this paper was to extend agent-based modeling towards the inclusion of intra-group dynamics. We emphasize that IM, UM, and IHM all account for the influence of social norms on household decision-making, which shows that modeling *inter*-scale interactions is not enough to explain social phenomena. Our inclusion of *intra*-scale dynamics allows us to not only model the cooperation and conflict present in a household, but the interaction between those dynamics and larger social norms. Although we selected gender in agriculture as a particularly compelling case for the need for intra-group models, our conclusions apply to the broader task of modeling social phenomena.

## Methods

### Overview of the intra-household model

Our conceptual framework (Fig. 2) links social norms ABMs with theoretical household models to simulate the interactions between individual, household, and population-level dynamics. Our framework can be used to design simulations which (a) run household models of bargains between agents and (b) maintain norms that comprise populations of individual agents. Thus, agent decision-making is constrained by both cooperation and conflict at the household level (lacking in both individual-based and unitary household ABMs) and by the pressure of social norms (lacking in traditional economic models). In addition, norms are affected by both individual preferences and household outcomes.

Figure 2 shows the modules that make up a simulation. Arrows indicate interaction between modules: Individual agents influence norms, norms influence individual agents, individual agents interact with one another through the household model, and activities influence individual agents’ decision-making. The household model takes two agents and executes a bargaining process each time step, for all agent pairs in a simulation. Agents bargain over how to spend their resources on activities, which are functions that take agent resources and characteristics and return goods. For example, a male agent might spend time on going to work and receive capital from a wage based on its gender; a female agent might do the same activity, but receive less capital. In more complex simulations, a trade network or crop model could be used as activity functions; in our example we used a simple wage.

Agents also make up the populations of norms. Norms have two attributes: A *population*, and a *protocol*. The population is the collection of agents that pay attention to the norm with more or less conformity. The protocol is the set of equations and statistics used to define the norm, e.g. the distance function used to calculate the difference between an agent’s behavior and the behavior of the rest of the population. Norms change based on the population behavior.

### The household model

Our household model draws heavily on Cudeville^15^. The household model executes a bargaining process between two agents over how to spend resources on activities. A *private activity* can be done by one agent and returns a private good, which can only be consumed by one agent (e.g. a job produces capital). A *public activity* can take resources from multiple agents and returns a public good, which can be consumed (shared) by multiple agents (e.g. housework produces a clean house). Private goods can be transferred between agents. Here we will show a model with one resource (time) and two activities (private and public) for simplicity.

Let a *bargain* consist of ($\alpha _A, \alpha _B, \gamma _A, \gamma _B, \theta _i$): The proportion of time Agent A spends on a private activity ($\alpha _A$), the proportion of time Agent B spends on a private activity ($\alpha _B$), the proportion of time A spends on a public activity ($\gamma _A$), the proportion of time B spends on a public activity ($\gamma _B$), and the transfer of some percentage of private good between A and B ($\theta _i$). Next, let $f_{\alpha }(), f_{\gamma }()$ be activity output functions which take agent input, in this case time, and return goods. For example, $f_{\gamma }(\gamma _A + \gamma _B)$ is a public activity function which takes time from both A and B and returns the same amount of public good to both. $f_{\alpha }^A(\alpha _A)$ is A’s private activity function, which may be different than B’s (e.g. different wages for the same job). For our simple demonstration, we used very straightforward functions: $f_{\alpha }(\alpha _{i}) = w_{i} \cdot \alpha _{i}$, where $i \in \{A,B\}$ and $w_{i}$ is a constant wage for A or B; and $f_{\gamma }(\gamma _{A} + \gamma _B) = \gamma _A + \gamma _B$ (see “Simulation experiments” section). Any functions can be used within our framework (for example, frequency-dependent functions that change output based on the number of agents that do activities).

Agents A and B have utility functions $U_A, U_B$ that take a bargain ($\alpha _A, \alpha _B, \gamma _A, \gamma _B, \theta _i$) and return utility via weights and output functions:

5 $$\begin{aligned} U_A =  [p_{\alpha }^A \sqrt{f_{\alpha }^A(\alpha _A) (1 - \theta _i)} + p_{\gamma }^A \sqrt{f_{\gamma }(\gamma _A + \gamma _B) }] \cdot e ^ {-N(...)} \end{aligned}$$

6 $$\begin{aligned} U_B =  [p_{\alpha }^B \sqrt{f_{\alpha }^A(\alpha _A) (\theta ) + f_{\alpha }^B(\alpha _B)} + p_{\gamma }^B \sqrt{f_{\gamma }(\gamma _A + \gamma _B)}] \cdot e ^ {-N(...)} \end{aligned}$$

Utility is a balance between personal preference and social pressure. Preferences *p* are weights for goods, e.g. $p_{\alpha }^A$ is how much Agent A likes private good, where $p_{\alpha }^A + p_{\gamma }^A = 1, p_{\alpha }^B + p_{\gamma }^B = 1$. Public goods are shared through $f_{\gamma }(\gamma _A + \gamma _B)$ and private goods are traded through $\theta _i$. In Eq. (5), $\theta _i$ percent of Agent A’s private good is removed, and in Eq. (6) it is added to Agent B’s private good. (The direction of transfer between A and B is determined by the bargaining process.)

The goods returned by $f_{\alpha }(),f_{\gamma }()$ are square-rooted to add restraint to an agent’s decision-making (i.e. an agent is unlikely to spend all her time dancing and never working, and similarly will not oscillate wildly in response to small changes in activity payoffs). The use of the square roots follow Cudeville, although we note again that the framework is function-agnostic and the user may designate their own utility functions^15^. Utility from personal payoff is then tempered by a *norm protocol* $e^{-N(...)}$, which we will return to in “Norms” section. We note that, although the notation suggests many parameters are needed for this model, the constraints show that the utility functions can be rewritten with fewer, like so: $U_A =  [p_{\alpha }^A \sqrt{f_{\alpha }^A(1 - \gamma _A) (1 - \theta _i)} + (1 - p_{\alpha }^A) \sqrt{f_{\gamma }(\gamma _A + \gamma _B) }] \cdot e ^ {-N(...)}$, assuming $\alpha _A + \gamma _A = 1$ and $\alpha _B + \gamma _B = 1$.

Maximizing Eqs. (5), (6) separately is not guaranteed to return the same selection of ($\alpha _A, \alpha _B, \gamma _A, \gamma _B, \theta _i$), because Agents A and B may have different preferences. In order to select a bargain for the household, we first define agent bargaining power and then formalize it in an objective function. Bargaining power is a person’s ability to affect household decisions, and is commonly referred to when discussing women’s empowerment^39^. Bargaining power can stem from multiple sources, including women’s physical assets, their human capital, laws and cultural/social norms. Following the convention of economic intra-household models, we define bargaining power as the utility of an agent when $\theta _i = 0$, the “separate spheres” approach^17^. We will write $\theta _i = 0$ as $\theta _s$. This definition can be formalized in the following objective function, which is maximized to select the best bargain $\theta _{max}$ for the household:

7 $$\begin{aligned} \mathop {\mathrm {argmax}}\limits _{\theta _i \in \theta } (U_A^{\theta _i} - U_A^{\theta _s}) (U_B^{\theta _i} - U_B^{\theta _s})\end{aligned}$$

8 $$\begin{aligned} (U_A^{\theta _i} \ge U_A^{\theta _s}), (U_B^{\theta _i} \ge U_B^{\theta _s}) \end{aligned}$$

where $U_A^{\theta _s}, U_B^{\theta _s}$ are Agents A and B’s utilities under $\theta _s$. In practice, we find the best bargain $(\alpha _A, \alpha _B, \gamma _A, \gamma _B, \theta _{max})$ in two steps. First, we converge on the intersection of A and B’s maximized utility functions through iterative optimization for multiple fixed $\theta _i$. For example, A selects a bargain that maximizes her utility; then B selects a bargain given A’s proposed level of $\gamma _A$; then A selects a bargain given B’s proposed level of $\gamma _B$; and so on until A and B agree on one bargain for some fixed $\theta _i$^40^. We then maximize Eq. (7) over all levels of $\theta _i$.

The bargaining process between Agents A and B can replicate household cooperation and conflict over almost anything, from who ought to do the housework to how far a woman is allowed to walk by herself. The implementation is flexible and dynamic, adjusting with each time step to changes in external factors such as wage while preserving important concepts from intra-household models such as bargaining power. The bargaining process also explicitly incorporates dynamics in behavioral norms, discussed in the next section.

### Norms

We introduced a norm protocol $e^{-N(...)}$ as a formalization of the influence of norms on agent utility functions. Here we unpack the norm protocol further, using Agent A and Norm Z as an example:

9 $$\begin{aligned} N(...) = N_Z(X) = \sum _i c_i^A D_Z(x_i^A, \zeta _i) \end{aligned}$$

where *X* is a set of components $x_1, x_2, ..., x_N$. A norm protocol is used by Agent A to gauge the distance $D_Z()$ between how Agent A behaves ($x_i^A$) and how the other agents in Norm Z behave ($\zeta _i$) at each time step. $D_Z()$ can be any measure of distance, $\zeta _i$ can be any comparison value, and *X* can contain any components, from the amount of time an agent spends on an activity to the preference an agent has for a good. For example, in our simulations we used the squared distance from the mean:

10 $$\begin{aligned} N_Z(\alpha _A, \theta _i) = \sum _{i = (\alpha _A, \gamma _A, \theta _i)} c_i^A(x_i^A - \mu _i)^2 \end{aligned}$$

for the amount of time an agent spends on private and public activities and the amount of capital transferred between spouses. $c_i^A$ is Agent A’s conformity parameter, i.e. how strong its preference is for following the norm.

### Comparison models

We compare our framework results to two of the most common types of ABMs found in the literature: Individual-based models of social norms and unitary household-based models.

Individual-based ABMs are often used to explore the emergence of norms and the effects of norms on individuals. Some individual-based ABMs have game theoretic bargains at their center and are about the creation and stability of norms^10,12^; others are primarily about the effects of existing and changing norms on agents^11,13^. We approximate the latter type of individual-based ABM by replacing Eqs. (5), (6), (7) with a single utility function maximized by an individual Agent A:

11 $$\begin{aligned} U_A =  [p_{\alpha }^A \sqrt{f_{\alpha }^A(\alpha _A)} + p_{\gamma }^A \sqrt{f_{\gamma }(\gamma _A) }] \cdot e ^ {- \sum _{i = (\alpha _A, \gamma _A)} c_i^A(x_i^A - \mu _i)^2} \end{aligned}$$

The second type of ABM is most often used to model land use and ecology^2,3,6,7^. Its basic unit of operation is the household, but it does not include a household model; household agents in these models are unitary, meaning that preferences and resources are assumed to be pooled and the “head” is the sole decision-maker. We approximate the unitary household ABM by having a single utility function for Household H, but retaining separate activity functions to preserve gender-based effects of external factors like wage for household members A and B:

12 $$\begin{aligned} U_H =  [p_{\alpha }^H \sqrt{f_{\alpha }^A(\alpha _A) + f_{\alpha }^B(\alpha _B)} + p_{\gamma }^H \sqrt{f_{\gamma }(\gamma _A + \gamma _B) }] \cdot e ^ {-\sum _{j = A, B} \sum _{i = (\alpha _j, \gamma _j)} c_i^j(x_i^j - \mu _i)^2} \end{aligned}$$

where the distance from the norm for each household member is summed for the household. It is worth pointing out that most unitary household ABMs do not incorporate social norms in this way. Throughout this paper we will refer to the comparison ABM using Eq. (11) as the “individual-based model” (IM) and the comparison ABM using Eq. (12) as the “unitary household-based model” (UM).

### Simulation experiments

We demonstrate our framework using some basic experiments and a scenario inspired by case studies of women’s empowerment in agriculture. Development groups often seek to empower women through economic interventions, investing in agricultural subsidies, technology and training to increase the payoff and decrease the cost of agricultural activities^29,31^. In a simulation, this might be formalized by increasing the output of an activity function $f_\alpha ()$ relative to its input. We constructed a scenario with two activities, one private and one public. Initially, the private activity returned wages of $w_f = 0.1, w_m = 0.6$ to female and male agents, respectively; the public activity returned the same amount of public good to both.

Simulations were first run until all populations reached a steady state. Then $w_m$ was held at 0.6 and $w_f$ was increased over one time step to $w_f = 0.15, 0.2, 0.25, 0.3, 0.4, ..., 1.0, 1.1$. That is, we explored the effects of a wage increase on agent behavior under conditions where the female wage was less than, equal to, greater than, or much greater than the male wage. We investigated smaller increases at $w_f < 0.3$ to emphasize differences between the intra-household model and the comparison models.

In addition to several levels of $w_f$, we tested the IHM, UM and IM under two conditions of goods preference and conformity: the “Moderate preferences” (MP) and “Conformist males” (CM) conditions. “Goods preference” refers to the weights assigned to goods in Eqs. (5), (6), (11) and (12) ($p_{\alpha }$ and $p_{\gamma }$). Agents have a weight for private good and a weight for public good. “Moderate” goods preference indicates that the weights were selected from a random uniform distribution $p_{i} \sim U(0.3,0.7)$ where $i \in \{\alpha , \gamma \}$. In both the MP and CM conditions, agents were parameterized with moderate goods preferences. However, the conformity of agents differed between conditions.

In the MP condition, agents were parameterized with conformity weights [$c_i$ in Eq. (9)] drawn from *U*(0.3, 0.7), i.e. the same distribution as the preferences. In the CM condition, conformity was consistently high for male agents, i.e. drawn from *U*(2.5, 3.0), but not for female agents. One interpretation of this parameterization might be that women receive empowerment training but men are ignored, leaving women to deal with their husband’s subscription to gender norms alone. For the CM condition, we ran simulations with parameterizations matching the MP condition until the wage change, at which point we increased the conformity of male agents at the same time as we increased the wage. This was to avoid very long convergence times pre-wage-change. In the unitary household parameterizations, all households were parameterized with high conformity at the wage change, rather than an equal number of high-conformity and moderate-conformity households; we did this because the unitary household model ignores intra-household preference in favor of a single household head, so the analogous parameterization to conformist framework males would be conformist households. We otherwise parameterized agents across model types as identically as possible (e.g., Agent 1 in IM had the same attribute values as Agent 1 in IHM).

200 agents were parameterized for each simulation, with agent attributes consistent across framework and comparison model simulations. Simulations were run once without repetition. All agents across all simulations were initialized at 80% private activity, 20% public activity for male agents and 20% private activity, 80% public activity for female agents, to mimic an existing norm of women spending most of their time doing household work. Male agents subscribed to one norm and female agents subscribed to another; that is, agents subscribed to a norm about the behavior of their own gender. Wage change occurred once all simulations had stabilized ($t = 25$ for our simulations) and the simulations were run until $t = 150$.
